# SG-APSIC1128: BHQ instrument management at Bangkok Hospital

**DOI:** 10.1017/ash.2023.101

**Published:** 2023-03-16

**Authors:** Rome Chomrak

**Affiliations:** Disinfection and Sterilization, Bangkok Hospital, Bankok, Thailand

## Abstract

**Objectives:** We evaluated the BHQ instrument management strategies at Bangkok Hospital as well as the general instrument and surgical instrument inventories to develop effective management of general and surgical instruments. **Methods:** A survey of instruments that had been used and were ready for use was conducted in all departments of Bangkok Hospital. Data were collected and analyzed using statistical methods to adapt the “refill and reduce” strategy. We determined usage rates from each department to determine inventory needs based on the principles that patients are safest with sufficient instruments available and that the central sterilization supply department (CSSD) can provide the best inventory management. **Results:** Our evaluation revealed that BHQ instrument management strategies can assist the hospital in reducing the cost of resterilizing instruments and thus can lower the workload and reduce tracking conflicts related to overdue instruments. **Conclusions:** This report confirmed earlier findings that Bangkok Hospital can have more instruments ready to use and can reduce costs without buying replacement instruments by using a strategy of “filling missing parts and cutting the excess.”

